# The Impact of Genome-Wide Supported Schizophrenia Risk Variants in the Neurogranin Gene on Brain Structure and Function

**DOI:** 10.1371/journal.pone.0076815

**Published:** 2013-10-02

**Authors:** Esther Walton, Daniel Geisler, Johanna Hass, Jingyu Liu, Jessica Turner, Anastasia Yendiki, Michael N. Smolka, Beng-Choon Ho, Dara S. Manoach, Randy L. Gollub, Veit Roessner, Vince D. Calhoun, Stefan Ehrlich

**Affiliations:** 1 Department of Child and Adolescent Psychiatry, University Hospital Carl Gustav Carus, Dresden University of Technology, Dresden, Germany; 2 The MIND Research Network, Albuquerque, New Mexico, United States of America; 3 Department of Electrical and Computer Engineering, University of New Mexico, Albuquerque, New Mexico, United States of America; 4 MGH/MIT/HMS Martinos Center for Biomedical Imaging, Massachusetts General Hospital, Charlestown, Massachusetts, United States of America; 5 Department of Psychiatry and Psychotherapy, University of Technology, Dresden, Germany; 6 Neuroimaging Center, Department of Psychology, University of Technology, Dresden, Germany; 7 Department of Psychiatry, University of Iowa, Iowa City, Iowa, United States of America; 8 Department of Psychiatry, Massachusetts General Hospital, Boston, Massachusetts, United States of America; United Graduate School of Child Development, Osaka University, Japan

## Abstract

The neural mechanisms underlying genetic risk for schizophrenia, a highly heritable psychiatric condition, are still under investigation. New schizophrenia risk genes discovered through genome-wide association studies (GWAS), such as *neurogranin* (*NRGN*), can be used to identify these mechanisms. In this study we examined the association of two common *NRGN* risk single nucleotide polymorphisms (SNPs) with functional and structural brain-based intermediate phenotypes for schizophrenia. We obtained structural, functional MRI and genotype data of 92 schizophrenia patients and 114 healthy volunteers from the multisite Mind Clinical Imaging Consortium study. Two schizophrenia-associated *NRGN* SNPs (rs12807809 and rs12541) were tested for association with working memory-elicited dorsolateral prefrontal cortex (DLPFC) activity and surface-wide cortical thickness. *NRGN* rs12541 risk allele homozygotes (TT) displayed increased working memory-related activity in several brain regions, including the left DLPFC, left insula, left somatosensory cortex and the cingulate cortex, when compared to non-risk allele carriers. *NRGN* rs12807809 non-risk allele (C) carriers showed reduced cortical gray matter thickness compared to risk allele homozygotes (TT) in an area comprising the right pericalcarine gyrus, the right cuneus, and the right lingual gyrus. Our study highlights the effects of schizophrenia risk variants in the *NRGN* gene on functional and structural brain-based intermediate phenotypes for schizophrenia. These results support recent GWAS findings and further implicate *NRGN* in the pathophysiology of schizophrenia by suggesting that genetic *NRGN* risk variants contribute to subtle changes in neural functioning and anatomy that can be quantified with neuroimaging methods.

## Introduction

Twin and family studies suggest a high heritability for schizophrenia [1], but results of candidate gene studies have been inconsistent. Often, findings could not be replicated and it is likely that genes unrelated to the canonical neurotransmitter pathways have an impact on disease etiology.

Genome-wide association studies (GWAS) allow for a hypothesis-free approach to genetic investigations. A recent study of more than 300,000 single nucleotide polymorphisms (SNPs) in 12,945 schizophrenia patients and 34,591 healthy controls reported associations at genome-wide significance with rs12807809 located near *neurogranin* (*NRGN*) [2]. Interestingly, the *NRGN* gene was also found to be associated with schizophrenia in three additional independent samples [3,4], although there have also been conflicting findings [5–7].

In order to understand the role of new genetic markers in disease pathophysiology careful clinical and biological follow up studies are necessary. Studying the effects of risk variants for psychiatric disorders on brain function and structure can provide insight into disease-associated changes and mechanisms on a neuroscience systems level, but also helps to verify GWAS results. Here, we studied the effects of *NRGN* risk variants on brain-based intermediate phenotypes for schizophrenia. Neuroimaging-based intermediate phenotypes are heritable, disease-associated and stable traits that may show a stronger association with risk genes than behavior or diagnosis due to their greater proximity to the underlying biology [8]. In fact, inconsistent findings in case-control studies can partially be due to small effect sizes of single genes on complex entities such as diagnostic categories. Dorsolateral prefrontal cortex (DLPFC) dysfunction during working memory processing and widespread reduced cortical thickness have both been shown to be heritable markers closely related to schizophrenia [9].

*NRGN* protein is an important component in the NMDA-signaling pathway, which is associated with synaptic plasticity and memory formation [10]. In fact, *NRGN* knockout mice display deficits in neural and behavioural correlates of learning and memory [11,12]. Furthermore, *NRGN* has been associated with working memory-elicited neural activity in healthy controls [13,14] while data from patient samples are still lacking. However, a study using postmortem brain tissue of schizophrenia patients found reduced *NRGN* immunohistochemical staining in working memory-associated areas such as the anterior cingulate cortex (ACC) and the DLPFC [15]. Furthermore, *NRGN* has been implicated in neuronal cortico- and synaptogenesis during brain development [16] – both found to be impaired in schizophrenia [17,18].

Given the evidence for an association of *NRGN* with a diagnosis of schizophrenia and cortical development, as well as a reduced *NRGN* expression in frontal brain regions of schizophrenia patients, the aim of the present investigation was to understand the neurogenetic risk mechanisms of two common *NRGN* SNPs (rs12807809 and rs12541) and their haplotypes by studying intermediate phenotypes for schizophrenia: abnormal working memory-elicited DLPFC activity and reduced cortical thickness. Because rostral ACC volume reduction, especially in the left hemisphere, has been associated with both, a diagnosis of schizophrenia and *NRGN* risk variants [19,20], we also investigated *NRGN* genotype effects on left rostral ACC volume in an additional analysis.

## Materials and Methods

### Participants

We studied a total of 206 participants (92 schizophrenia patients and 114 healthy volunteers) who enrolled in the multisite Mind Clinical Imaging Consortium study [21,22], were between 18 and 60 years of age, and fluent in English and who had complete structural, functional MRI and genotype data. Patients had a Diagnostic and Statistical Manual of Mental Disorders (*DSM-IV*) diagnosis of schizophrenia, established using a Structured Clinical Interview for DSM disorders (SCID) and a review of case files by trained clinicians. There were no exclusions based on treatment with antipsychotic drugs. For further details, see SI 1.1 in File S1 and [23].

Controls were matched to the patient cohort for age, gender, and parental education and were excluded if they had a history of a medical or Axis I psychiatric diagnosis. Participants were excluded if they had a history of neurological or psychiatric disease other than schizophrenia, history of a head injury, history of substance abuse or dependence within the past month, severe or disabling medical conditions, contraindication to MR scanning or an estimated verbal IQ less than 70 (based on the reading subtest from the Wide Range Achievement Test (WRAT-III)).

### Ethics statement

After complete description of the study the participants provided written informed consent. The human research review committees at each of the four sites (Universities of Iowa (UI), Minnesota (UMN), and New Mexico (UNM) and Massachusetts General Hospital (MGH)) approved the study protocol. We confirm that all potential participants who declined to participate or otherwise did not participate were eligible for treatment (if applicable) and were not disadvantaged in any other way by not participating in the study. During the consent process the subjects were asked a series of questions to assure that they understood the nature of the study, that if they chose to participate it was voluntary and that they could stop at any time without affecting their care, and that they understood the risks and benefits of the study. If they stated that they wanted to participate, they were also asked the reason why they chose to participate. If there was any question as to the ability to provide informed consent (i.e., they don’t understand the risks or benefits, or they suffer from acute delusions that could significantly impair a patient’s judgment) then they were not recruited for the study. In addition, if during the clinical interview it was determined that they lacked the ability to provide informed consent, then they were dropped from the study at that time.

### Behavioral task

The Sternberg Item Recognition Paradigm (SIRP) is a working memory task, previously shown to consistently activate the DLPFC and parietal regions in healthy controls and schizophrenia patients [24]. In each block during the Encode phase, a memory set, composed of one (load 1), three (load 3), or five (load 5) digits, was presented (two blocks per load condition). The Encode phase was followed by a presentation of 14 digits, one at a time (the Probe phase) and participants responded to each probe to indicate whether or not the probe digit was in the memory set. For additional details about the paradigm, see [21] and SI 1.2 in File S1.

### Image acquisition and processing

Structural magnetic resonance imaging (MRI) data was acquired with either a 1.5T Siemens Sonata (UNM, MGH, UI) or a 3T Siemens Trio (UMN). Functional MRI data was acquired with either a 1.5T Siemens Sonata (UNM) or a 3T Siemens Trio (UMN, MGH, UI). To reduce variability due to acquisition site differences, all sites followed guidelines developed by the biomedical informatics research network (BIRN) test bed, which included standardized acquisition parameters across sites (matched button press devices, common calibration methods, usage of human phantoms) [25,26].

Cortical reconstruction and volumetric segmentation based on high resolution structural MRI scans was performed with the FreeSurfer surface reconstruction software (http://surfer.nmr.mgh.harvard.edu, for more details, see SI 1.3 in File S1). Functional data were analyzed using the Function Biomedical Informatics Research Network (FBIRN) Image Processing Stream (FIPS), a pipeline using the Functional MRI of the Brain (FMRIB) Software Library of FSL (http://www.fmrib.ox.ac.uk/fsl). For additional information about data acquisition and processing, see SI 1.3 in File S1.

### Genotyping

Blood samples were obtained from 255 participants and sent to the Harvard Partners Center for Genetics and Genomics for DNA extraction. All DNA extraction and genotyping was done blind to group assignment. Genotyping was performed at the Mind Research Network Neurogenetics Core Lab using the Illumina HumanOmni-Quad BeadChip. Quality control steps included common standard procedures [27] using PLINK, 1.06 [28]. We removed seven participants with extreme heterozygosity values (+/- 3SD) resulting in a final dataset of 206 participants after excluding additional participants failing imaging quality control steps (see SI 1.3 in File S1). Using this dataset, *NRGN* SNPs rs12541 and rs12807809 were extracted. The SNP rs12807809 was reported to be significantly associated with schizophrenia in a recent GWAS [2]. Based on this, we searched for other potential disease-associated SNPs in the *NRGN* gene using the continuously updated meta-analysis of genetic studies on schizophrenia available at http://www.schizophreniaresearchforum.org updated on October 22^nd^, 2010. Apart from the above mentioned SNP rs12807809, this website lists another three SNPs for *NRGN*: rs7113041, rs1804829, and rs12541. Our datasets contained rs12807809 and rs12541. Since it is not uncommon that different genetic studies identify the same risk genes while their results differ in the risk allele structure, we used data from the most recent and largest GWAS [29] including 6,458 schizophrenia cases and 8,971 controls, to identify the risk allele. According to this dataset the T allele represents the risk allele for both rs12807809 (p=0.020) and rs12541 (p=0.017). The two SNPs were not in linkage disequilibrium with each other (r^2^ = 0.05). More quality control measures are given in Table S1 in File S1. Because of the low frequency of C/C genotypes for both rs12541 and rs12807809 (n_rs12541_=17, n_rs12807809_=8), C/C and C/T participants were combined into one C allele-carriers group (n_rs12541_=87, n_rs12807809_=85). We hypothesized that TT allele homozygosity in both SNPs would be associated with abnormal working memory-elicited DLPFC activity and reduced cortical thickness.

### Statistical analyses

Basic demographic characteristics were compared across genotype group and all four acquisition sites using a series of one-way ANOVA and subsequent Bonferroni-corrected post hoc tests. Chi-square statistics were used to examine differences in categorical variables. Alpha was set to 0.05 for all analyses.

In our fMRI analyses, we used a Contrast Of Parameter Estimate (COPE) that modeled all working memory loads (load 1, load 3, load 5) during the Probe phase versus fixation. In our main higher level models (referred to as model 1 for rs12541 and model 2 for rs12807809) we tested the effects of genotype of each SNP by fitting a univariate general linear model to the fMRI time course at each voxel in the whole brain to estimate the average activation during the three loads of the probe condition in a whole brain model. Equal weight was given to all loads. All models were cluster-corrected according to FSL default settings (following random field theory) with a z-value of 2.3 and a p-value of 0.05 and controlled for scanner field strength and diagnostic group. We also modeled the diagnosis by SNP interaction effect.

In order to control for potentially confounding effects of population stratification, we checked rs12541 and rs12807809 allele frequencies across population groups. Hapmap3 data (http://hapmap.ncbi.nlm.nih.gov) showed that individuals of African ancestry (Hapmap populations: African ancestry in Southwest USA (ASW), Luhya in Webuye, Kenya (LWK), Maasai in Kinyawa, Kenya (MKK), and Yoruban in Ibadan, Nigeria (YRI)) had a much higher rs12541 C allele frequency than all other populations (Chi-Square test χ^2^=27.592, p<0.001) (Table S2 in File S1). This could be confirmed in our own sample (Chi-Square test χ^2^=19.512, p<0.001). No allele frequency differences were observed for rs12807809 (Chi-Square test_Hapmap_ χ^2^=1.333, p=0.248; Chi-Square test_MCIC_ χ^2^=2.667, p=0.102). We therefore tested the effect of rs12541 genotype in an additional model (model 1a) on a sample limited to participants of non-African ancestry (n=182).

Entire cortex vertex-wise analyses of cortical thickness were performed contrasting rs12541 and rs12807809 C allele carriers vs. TT homozygotes. Briefly, spherical registered cortical thickness data from all subjects were mapped to an average subject (http://surfer.nmr.mgh.harvard.edu/fswiki/FsAverage). Cortical thickness maps were smoothed using a 10mm full-width-at-half-maximum Gaussian kernel. Finally, univariate general linear models were run for each SNP separately (model 3 and 4) at all vertices (n=163,842) per hemisphere. We included age, gender, scanner field strength and diagnostic group into the models as covariates and also tested for the diagnosis by SNP interaction effect. All cortical thickness results were corrected for multiple comparisons using a Monte-Carlo simulation with 10,000 repeats. Vertex-wise threshold and cluster-wise probability (CWP) were set to 0.05. For details, see SI 1.6 in File S1. Final statistical maps are shown on the inflated surface of the standard average subject, allowing visualization of data across the entire cortical surface without interference from cortical folding.

Given previous results indicating an association between *NRGN* rs12807809 and left rostral ACC volumes [19,20] we tested this relationship in an additional analysis (model 4a) using a two-way ANCOVA model in SPSS (SPSS Inc., Chicago IL) with the two genotype groups and diagnostic group as factors as well as age, gender, scanner field strength, and intracranial volume as additional covariates of no interest. Total intracranial volume [30] and rostal ACC volume are a standard output of the FreeSurfer volumetric segmentation [31].

In order to perform haplotype subanalyses on our main findings, we obtained estimates of the actual cortical thickness in the identified cluster in millimeter (mm) and indices of neural activity for the DLPFC in mean percent signal change (mean %Δ). Haplotype analyses were carried out in Plink. In detail, a standard E-M algorithm was used to impute the distribution of probabilistically-inferred sets of haplotypes for each individual. Then we carried out a linear regression omnibus test with three degrees of freedom, jointly testing all haplotype effects on both intermediate phenotypes (model 5 and 6), covarying for the effects of diagnosis and scanner field strength and additionally for age and gender in the structural model. In case of a significant omnibus test, we carried out haplotype-specific tests to infer the direction of the effects.

To check for potential medication effects, we used the extracted activation and thickness estimates, regressed out all relevant covariates and correlated these residuals with lifetime exposure to antipsychotic medication estimates. Furthermore, we tested the effects of *NRGN* in schizophrenia patients and healthy individuals in separate analyses. Statistical analyses were carried out in SPSS 17.0. Power analyses were carried using G*Power 3 [32]. Additional details are included in SI 1.7, SI 1.8, and SI Figure S1 in File S1.

## Results

### Sample characteristics

Patients and controls did not differ in age, parental socio-economic status (SES) or handedness, but the percentage of females and participants of European descent among the healthy controls was higher and patients had lower WRAT-IIIRT Scores. There was no effect of acquisition site on gender, WRAT-IIIRT Score or handedness, but sites differed in their participants’ age, parental SES and ancestry (Table 1).

**Table 1:** Basic demographics according to acquisition site.

Site	Sample	Size	Gender (female)		Ancestry (White/African)^c^		Age	WRAT-IIIRT	Parental SES	Handedness	Cumulative antipsychotic drug dose^d^	Current antipsychotic drug dose^e^	Negative symptoms	Positive symptoms	Duration of illness
		N	N	%	N	%	Mean (SD)	Mean (SD)	Mean (SD)	Mean (SD)	Mean (SD)	Mean (SD)	Mean (SD)	Mean (SD)	Mean (SD)
MGH	SCZ	25	7	28	16/7^b^	64.0/28.9	37.92^b^ (9.81)	45.09 (8.49)	3.40^b^ (1.12)	0.61 (1.92)	98.19 (210.11)	519.38 (489.85)	6.96 (4.46)	4.56 (3.31)	15.43 (11.85)
	HC	23	10	43.5	14/4^b^	60.9/17.4	40.04^b^ (9.59)	51.96 (3.98)	3.00^b^ (0.95)	1.04 (2.93)	-	-	-	-	-
UI	SCZ	22	3	13.6	21/1^b^	95.5/4.5	31.81^b^ (8.91)	48.38 (5.04)	2.33^b^ (0.66)	0.82 (2.81)	44.17 (57.48)	617.86 (644.45)	9.32 (4.24)	4.23 (2.99)	9.05 (6.47)
	HC	52	25	48.1	50/0^b^	96.2/0.0	30.24^b^ (10.46)	50.08 (4.07)	2.87^b^ (0.44)	0.69 (2.57)	-	-	-	-	-
UMN	SCZ	27	8	29.6	19/6^b^	70.4/22.2	31.63^b^ (10.63)	46.22 (5.43)	2.52^b^ (0.75)	1.78 (3.59)	33.40 (77.66)	662.19 (955.05)	7.33 (2.88)	5.00 (2.47)	9.06 (8.56)
	HC	17	7	41.2	17/0^b^	100.0/0.0	31.12^b^ (11.30)	50.94 (4.09)	2.35^b^ (0.79)	0.47 (0.80)	-	-	-	-	-
UNM	SCZ	18	5	27.8	17/0^b^	94.4/0.0	35.83 (14.09)	45.53 (7.05)	2.88^b^ (1.15)	1.39 (3.13)	24.14 (32.01)	632.35 (525.80)	8.78 (3.57)	4.72 (2.74)	11.08 (12.58)
	HC	22	4	18.2	21/0^b^	95.5/0.0	30.81 (12.90)	51.50 (3.79)	2.14^b^ (0.77)	1.05 (2.42)	-	-	-	-	-
Total	SCZ	92	23^a^	25	73/14^a^	79.3/15.2	34.23 (11.01)	46.31^a^ (6.60)	2.79 (1.01)	1.17 (2.94)	50.93 (120.70)	606.90 (650.41)	7.99 (3.89)	4.64 (2.86)	11.18 (10.23)
	HC	114	46^a^	40.4	102/4^a^	89.5/3.5	32.49 (11.44)	50.86^a^ (4.02)	2.68 (0.76)	0.80 (2.43)	-	-	-	-	-

A series of linear and logistic regression as well as chi-square analyses were performed to detect significant differences of age, WRAT-IIIRT Score, parental SES, handedness, gender and ancestry between acquisition sites and diagnostic groups. Abbreviations: WRAT-IIIRT, reading subtest of the Wide Range Achievement Test - III; SES, socio-economic status; handedness, Annett Handedness Scale; MGH, Massachusetts General Hospital; UI, University of Iowa; UMN, University of Minnesota; UNM, University of New Mexico; SCZ, schizophrenia patients (202 patients diagnosed with schizophrenia, three patients with schizophreniform and one patient with schizoaffective disorder, established using a Structured Clinical Interview for DSM disorders (SCID) [70] and a review of case files by trained clinicians); HC, healthy controls. ^a^ significantly different between SCZ and HC on the basis of a chi-square test, or a linear or logistic regression (*p*<0.05); ^b^ significantly different between acquisition sites on the basis of a chi-square test, or a logistic or a linear regression with subsequent Bonferroni post hoc tests (*p*<0.05); ^c^ ancestry based on self report, numbers not shown for other ancestries, mixed descent or missing data; ^d^ excluding two patients, who had never been on antipsychotic medication; ^e^ excluding three patients, who were at the time of the study not on antipsychotic medication.

For both *NRGN* SNPs, rs12541 and rs12807809, there were no differences between genotype groups with respect to diagnosis, gender, age, WRAT-IIIRT Score, parental SES, handedness, working memory performance, reaction time, and acquisition site (Table 2). The percentage of participants of European descent among the rs12541 TT homozygotes was higher than for C carriers.

**Table 2:** Basic demographics according to *NRGN* rs12541 and rs12807809 genotype.

	Diagnosis (SCZ Patients)		Sex (female)		Ancestry (White/African)^a^		Age	WRAT-IIIRT	Parental SES	Handedness	Performance	Reaction time	Site (MGH/UI/UMN/UNM)
	N	%	N	%	N	%	Mean (SD)	Mean (SD)	Mean (SD)	Mean (SD)	Mean (SD)	Mean (SD)	N
rs12541													
C carriers (n=87)	39	44.8	32	36.8	68/14	78.2/16.1	34.23 (10.97)	48.61 (6.54)	2.73 (0.96)	1.25 (3.01)	96.84 (4.15)	678.95 (97.64)	28/30/15/14
TT homozygotes (n=119)	53	44.5	37	31.1	107/4	89.9/3.4	32.55 (11.46)	49.08 (5.10)	2.72 (0.81)	0.74 (2.37)	97.44 (2.82)	676.99 (117.60)	20/44/29/26
χ^2^/t-test	0.002		0.730		10.215		-1.052	0.554	-0.117	-1.308	-1.233	-0.102	7.240
df	1		1		2.0		201	158.67	201	163.58	201	129	3
p	0.967		0.393		0.006		0.294	0.580	0.907	0.909	0.219	0.919	0.065
rs12807809													
C carriers (n=85)	36	42.4	26	30.6	68/10	80.0/11.8	33.63 (11.53)	48.38 (6.02)	2.80 (0.87)	0.93 (2.30)	97.22 (3.43)	688.04 (116.95)	19/26/22/18
TT homozygotes (n=121)	56	46.3	43	35.5	107/8	88.4/6.6	33.02 (11.10)	49.23 (5.55)	2.68 (0.88)	0.98 (2.90)	97.15 (3.49)	669.75 (102.27)	29/48/22/22
χ^2^/t-test	0.312		0.549		2.784		-0.382	1.033	-0.962	0.142	0.15	-0.954	2.819
df	1		1		2.0		201	200	201	202	201	129	3
p	0.577		0.459		0.249		0.703	0.303	0.337	0.887	0.881	0.342	0.420

WRAT-IIIRT, reading subtest of the Wide Range Achievement Test – III; SES, socio-economic status; handedness, Annett Handedness Scale. ^a^ ancestry based on self report, numbers not shown for other ancestries, mixed descent or missing data. Due to measuring device errors at one acquisition site, reaction time data is based on 70 SCZ and 61 HC. T-tests did not show any significant main effects of rs12541 or rs12807809 genotype group (C carrier vs. TT) on age, WRAT-IIIRT Score, parental SES, handedness, working memory performance, and reaction time. Chi-square statistics did not reveal any relationships between genotype and diagnosis, gender or acquisition site, but there was an effect of ancestry on rs12541 genotype groups.

### Functional MRI

**Figure 1:**
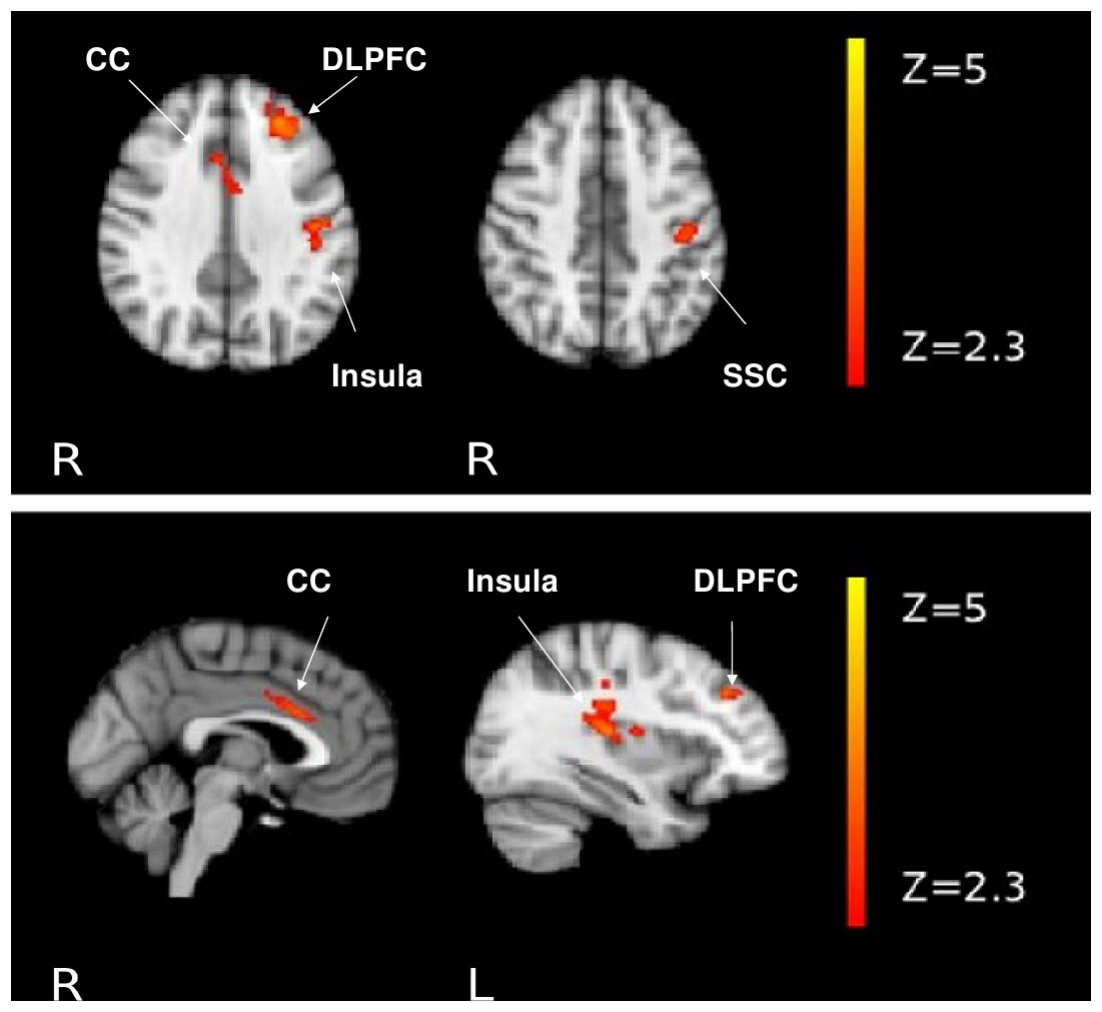
Effect of *NRGN* risk variant on brain function. Functional map illustrating increased neural activity in rs12541 TT homozygotes compared to C carriers. SSC, somatosensory cortex; CC, cingulate cortex. Results were cluster-corrected and z-values are represented according to the color code.

Main effects of task on activation were observed in working memory-associated brain regions such as the DLPFC and parietal regions as described previously [22,33]. *NRGN* rs12541 TT homozygotes displayed increased working memory-related activity in several brain regions when compared to C carriers in a whole-brain model (model 1) covarying for the effects of scanner field strength and diagnosis. Local maxima were found in areas including the left DLPFC, left insula, left somatosensory cortex and the cingulate cortex (Figure 1). Cluster-related maxima were found over the left DLPFC and the left insula. For more details on the statistical assessment of each of these findings, see SI 2.1 and SI Table S3 in File S1. We found no increased neural activity in C carriers compared to TT homozygotes. Also, the interaction term between *NRGN* rs12541 genotype and diagnosis was not significant. In subanalyses, investigating patients and controls separately, we found comparable genotype effects on DLPFC dysfunction in each group. Furthermore, we did not find a significant correlation with lifetime exposure to antipsychotic medication. Neural activity did also not differ by rs12807809 genotype (model 2).

In an additional whole-brain model (model 1a) excluding participants of African ancestry from the analysis (remaining sample size n=182, see methods section for further details) and again controlling for the same covariates, the main finding from our initial model could be replicated, i.e. TT homozygotes had increased neural activity in the left DLPFC, left insula, left somatosensory cortex and the cingulate cortex when compared to C carriers (Figure S2 in File S1). Cluster-related maxima were found over the left DLPFC and the cingulate cortex. Again, no significant increase of activity of C carriers compared to TT homozygotes and no interaction between *NRGN* rs12541 and diagnosis could be found.

Additional haplotype analyses (model 5) showed that DLPFC activity was higher in participants with the *NRGN* rs12541-rs12807809 TT haplotype and lower for CC and CT haplotypes confirming *NRGN* rs12541 T allele as the major risk allele (see SI 2.3 and Table S4 in File S1).

### Structural MRI

**Figure 2:**
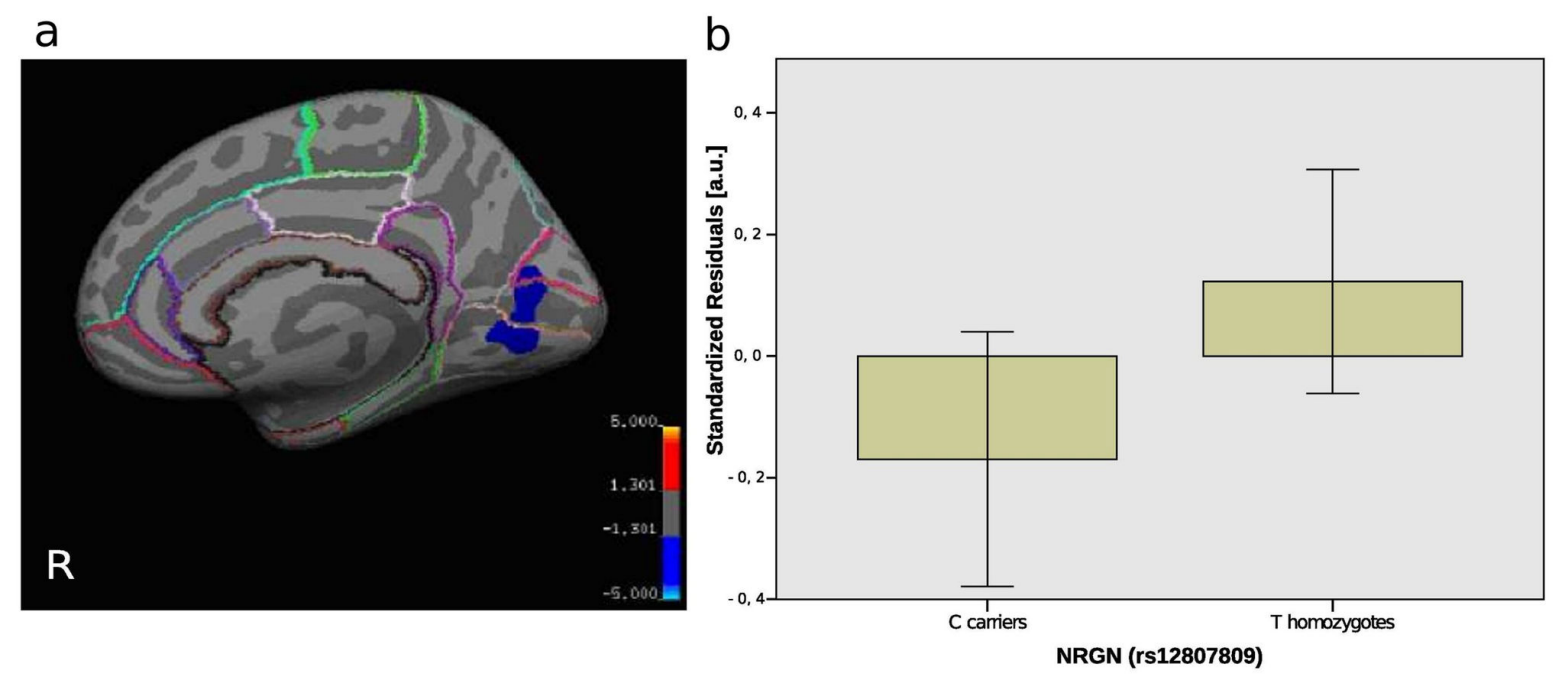
Effect of *NRGN* risk variant on cortical thickness and ACC volume. a) Cortical statistical map illustrating reduced cortical thickness for rs12807809 C carriers compared to TT homozygotes. The -log(CWP-value) is represented according to the color code. b) Boxplot showing mean and two standard errors of the standardized residuals for the effects of *NRGN* rs12807809 genotype on left rostal ACC volume controlled for intracranial volume, age, gender, diagnosis and scanner field strength.

In accordance with previous findings we observed widespread bilateral thickness reductions in schizophrenia patients (data not shown, for details please refer to [33]). *NRGN* rs12807809 C carriers showed reduced cortical gray matter thickness compared to TT homozygotes in an area comprising the right pericalcarine gyrus, the right cuneus, and the right lingual gyrus in an entire surface model (model 4) controlling for gender, diagnosis, age and scanner field strength (Figure 2a, corrected for multiple comparisons). The average thickness in this cluster of TT homozygotes (1.84 mm) was reduced by 3.88% in C carriers (1.77 mm). Again, the interaction term between *NRGN* rs12807809 and diagnosis was not significant. In subanalyses investigating patients and controls separately, we found qualitatively similar genotype effects on cortical thickness. There was no effect of rs12541 genotype on cortical thickness (model 3) and no effect of lifetime exposure to antipsychotic medication on cortical thickness in the cluster identified in our surface-wide analysis.

Additional haplotype analyses (model 6) confirmed *NRGN* rs12541-rs12807809 TC as the risk haplotype (β=-0.0669; *p*=0.0144) supporting our main findings that identified the C allele in rs12807809 as a potential risk allele (see SI 2.3 and Table S4 in File S1).

Additional analysis (model 4a) with a region-of-interest approach revealed that *NRGN* rs12807809 C carriers displayed reduced left rostral ACC volume compared to TT homozygotes (Figure 2b, F(1,194)=3.99, *p*=0.047). The average rostral ACC volume was reduced by 6.32% in C carriers (2325.95 mm^3) when compared to TT homozygotes (2482.85 mm^3). Again, no significant interaction between *NRGN* rs12807809 and diagnosis was found.

## Discussion

In the present study we used multimodal brain imaging to explore potential effects of the schizophrenia risk gene *NRGN* on two independent schizophrenia-related brain-based intermediate phenotypes in schizophrenia patients and healthy controls. We found that NGRN rs12541 TT homozygotes showed increased neural activity during a working memory task in the left DLPFC and other task-associated areas such as the ACC and the left insula. *NRGN* rs12807809 C carriers showed regional cortical thinning in the right pericalcarine gyrus, the right cuneus, and the right lingual gyrus as well as reduced left ACC volume. Haplotype analyses further supported the T allele in rs12541 and the C allele in rs12807809 as potential risk alleles.

These results support recent findings from a GWA study and other independent case-control studies, which found associations between *NRGN* and schizophrenia [2–4]. Given that some case-control studies also reported negative findings for *NRGN* [5–7] - possibly due to small effect sizes of single genes on complex, polygenic and heterogeneous clinical phenotypes - our results also underline the importance of follow-up studies using brain-based intermediate phenotypes to investigate the mechanisms of new genetic markers on a neuroscience systems level.

DLPFC dysfunction during working memory is a widely acknowledged intermediate phenotype for schizophrenia [9]. Compared to performance-matched healthy controls, patients display aberrant DLPFC functioning across task difficulties [34] by recruiting more neural resources for easy tasks (hyperfrontality, often seen with the SIRP task [24,34–36]), but may show decreased frontal neural activity (hypofrontality) and declining behavioral performance when the task is too difficult [35,37]. This pattern has been termed “inefficiency” of the prefrontal cortex [24,34,35]. DLPFC dysfunction can also be observed in medication naïve schizophrenia patients, high risk individuals and those showing prodromal symptoms [34,38–40] and therefore reflects most likely a medication independent process.

As part of a larger working memory network the ACC and the insula are also involved in many cognitive and attention-related processes [41,42]. Aberrant ACC and insula activity in schizophrenia patients has been observed during the SIRP working memory paradigm [43–45], during the n-back working memory task [46] and during attentional and executive tasks [47–49], although the direction of the effect varied, depending on the difficulty of the task.

Associations between *NRGN* and a functionally defined brain-based schizophrenia intermediate phenotype is in line with the aforementioned GWA and post-mortem brain tissue findings [15]. A study by Krug et al. [13] also found effects of *NRGN* variants on brain activation. In their study, healthy controls had to memorize neutral faces for a later recognition trial. *NRGN* rs12807809 risk allele TT homozygotes had increased neural activity in the ACC during the encoding phase and less deactivation in the left insula during the recognition phase. Similarly, Rose et al. [14] investigated *NRGN* genotype effects on brain activity during a spatial working memory task in healthy controls. They reported a failure to disengage ventromedial prefrontal areas in rs12807809 risk allele TT homozygotes, but found no effect on grey or white matter volume. Despite discrepancies with respect to risk SNP and allele (which might be due to differences in the functional paradigms and sample characteristics), our joint findings suggest that *NRGN* may be involved in biological pathways, which eventually affect a broader working memory network including the DLPFC.

What are the potential molecular mechanisms of *NRGN* risk variants? NRGN binds CaM and thereby acts as a regulator of downstream CaM-associated pathways which include AMPA receptor insertion into the postsynaptic membrane [50–52]. A disturbance of AMPA receptor homeostasis might impair NMDA receptor-mediated mechanism of working memory [53,54]. Thus, a possible downstream effect of *NRGN* dysfunction could be an imbalance of different glutamate channels impeding working memory processes.

Furthermore, *NRGN* was also associated with cortical thinning in three adjacent occipital areas (right pericalcarine gyrus, right cuneus, and right lingual gyrus). Many studies have reported cortical thickness reduction in occipital areas of schizophrenia patients [55,56], although there is some heterogeneity with respect to the size of the effect [57–60]. Heritability estimates of around 0.55 were reported for occipital areas in healthy pedigrees [61]. Cortical thickness is assumed to reflect the arrangement and density of neuronal and glial cells as well as passing axons shaped in early brain development and later pruning processes [62]. Postmortem studies in schizophrenia patients report abnormalities in neuronal migration as well as reductions in neuronal size and arborization compared to healthy control brains [17,18]. *NRGN* has been suggested to play a role in the arborization processes and synaptogenesis in early development. Studies in macaque monkeys suggest that *NRGN* expression levels peak in early development at around postnatal day 70 [16]. This period roughly coincides with a maximum burst of synaptogenesis in monkeys around the second postnatal month [63]. Moreover, in developing monkeys, the earliest and highest *NRGN* expression occurred in primary visual areas in the occipital lobe. In adult monkeys *NRGN* expression levels differ substantially between occipital and other neocortical layers [64]. It is therefore possible that neuronal corticogenesis, especially in occipital areas, is susceptible to *NRGN* disturbances during development.

Interestingly, Ohi et al. [20] reported an association between *NRGN* rs12807809 TT homozygotes and reduced gray matter volume in the ACC in a group of schizophrenia patients of Japanese ancestry but not in controls. Similarly, we found an association between ACC volume and *NRGN* rs12807809 in our additional analysis. However, in our study C allele carriers had reduced volumes. It is not uncommon that the risk allele structure is inconsistent when comparing different studies and possible reasons for this phenomenon might be multilocus effects, variation in local patterns of LD, population structure of study samples (the Ohi et al. sample was of Japanese ancestry), and environmental exposure differences between study populations [65–67]. Taken together, our joint findings points towards an involvement of *NRGN* in brain development.

Given the reported relationship between the *NRGN* gene and risk for schizophrenia, the present results suggest that the studied risk variants may contribute to disease risk via increased DLPFC activation (i.e., inefficiency) and decreased cortical thickness as well as brain volumes in specific brain regions. However, the results of the current study indicate that the effect of these genotypes on brain-based phenotypes is not limited to schizophrenia patients. It is possible that other common polymorphisms, rare variants, epigenetic or environmental factors interact with the investigated *NRGN* polymorphisms and enhance the adverse effect of these variants in vulnerable individuals. If we consider that the investigated *NRGN* variants were previously associated with schizophrenia and that DLPFC dysfunction and reduced cortical thickness are well validated intermediate phenotypes for schizophrenia, our imaging genetics results support a robust relationship between *NRGN* and schizophrenia.

The findings of our study have to be interpreted in the light of the following limitations. First, we focused on two *NRGN* SNPs that have been previously associated with schizophrenia. However, it is also possible that these SNPs are in high linkage disequilibrium with other functional variants which represent the true underlying genetic determinants responsible for the effects described in our paper. Second, the studied SNPs were associated with different brain modalities and the presumably protective allele of rs12807809 (as identified in Ripke et al. [29]) was related to decreased cortical thickness and ACC volume. These findings may indicate that the effect of *NRGN* on brain structure and function is more complex and that incomplete penetrance, epistasis, pleiotropy, imprinting and genetic heterogeneity may play a role. Given that both SNPs are not in linkage disequilibrium with each other, it is unsurprising that they may impact gene function and thus the intermediate phenotypes somewhat differently. However, considering the significant impact of *NRGN* haplotypes on both intermediate phenotypes, it is also possible that this study was simply underpowered to detect the presumably weaker effects of the respective other SNP on our two major intermediate phenotypes. Third, the associations between *NRGN* and both brain function and cortical thickness in schizophrenia patients may be influenced by the effects of antipsychotic medications. Despite our attempts to estimate the influence of antipsychotics (which indicated no effects on our results), we are currently unable to distinguish completely between the potential effects of antipsychotic medications versus those of the underlying disease process on measures of brain function and structure. However, prefrontal dysfunction and reduced cortical gray matter thickness have been shown to occur in persons with a high risk of developing schizophrenia and among neuroleptic-naïve and very young patients with a first episode of schizophrenia [40,68]. Furthermore, a study investigating the effect of antipsychotic medication on cortical thickness failed to find an association [69]. This implies that the reported associations are likely to be medication-independent.

## Conclusions

Taken together, our study highlights the effects of schizophrenia risk variants in the *NRGN* gene on brain-based intermediate phenotypes for schizophrenia – DLPFC inefficiency during a working memory task and reduced cortical thickness. These results further implicate *NRGN* in the pathophysiology of schizophrenia and suggest that genetic *NRGN* risk variants contribute to subtle changes in neural functioning and anatomy which can be quantified with neuroimaging methods. 

## Supporting Information

File S1 **Supporting Material and Methods, Results.** Table S1. Quality control measure for rs12807809 and rs12541. Table S2, Allele frequencies across populations. Table S3, Results of functional and structural imaging models for rs12541 and rs12807809 respectively. Table S4, rs12541-rs12807809 haplotype analysis results. Figure S1, Power analysis of fMRI models. Figure S2, Additional Model.
(PDF)
