# Supplementary material for: The Impact of Genome-Wide Supported Schizophrenia Risk Variants in the Neurogranin Gene on Brain Structure and Function
Source: PLoS One. 2013 Oct 2;8(10):e76815. doi: 10.1371/journal.pone.0076815 (PMC3788740; doi:10.1371/journal.pone.0076815)
Supplement: File S1 — Supporting Material and Methods, Results. Table S1. Quality control measure for rs12807809 and rs12541. Table S2, Allele frequencies across populations. Table S3, Results of functional and structural imaging models for rs12541 and rs12807809 respectively. Table S4, rs12541-rs12807809 haplotype analysis results. Figure S1, Power analysis of fMRI models. Figure S2, Additional Model. (PDF) [file pone.0076815.s001.pdf]

Supporting Information for:

The impact of genome-wide supported schizophrenia risk variants in the neurogranin gene on brain structure and function

Esther Walton, M.Sc., Daniel Geisler, M.Sc., Johanna Hass, M.Sc., Jingyu Liu, Ph.D., Jessica Turner, Ph.D., Anastasia Yendiki, Ph.D., Michael Smolka, M.D., Beng-Choon Ho, M.D., Dara S. Manoach, Ph.D., Randy L. Gollub, M.D., Ph.D., Veit Roessner, M.D., Vince D. Calhoun, Ph.D. and Stefan Ehrlich, M.D.*

* Email: transden.lab@uniklinikum-dresden.de

SI 1.1 Antipsychotic history

Antipsychotic history was collected as part of the psychiatric assessment using the PSYCH instrument [1], and cumulative and current antipsychotic exposure was calculated using the chlorpromazine (CPZ) conversion factors of Woods et al. [2]. To calculate cumulative dose years, the following formulas were applied:

Cumulative dose years=[(Dose in mg/day) * (Days on dose)] / [conversion factor *(365.25 days)]

For current antipsychotic exposure, we converted all current doses into chlorpromazine units, using the formula below:

Chlorpromazine units=[(Dose in mg/day) / (Conversion factor)] * [100 CPZ units/mg]

SI 1.2 Sternberg item recognition paradigm

Sternberg Item Recognition Paradigm (SIRP). For each task block, participants were presented with a prompt, ‘‘Learn’’, and then following a 0.5 s delay, they were shown a memory set comprised of one, three, or five digits for 6 s. This was followed by a ‘‘probe epoch’’, which lasted 38 s, and consisted of a series of 14 probe digits presented for 1.1 s with a jittered intertrial interval of ≤1.6 s. Participants used a button box to indicate whether each probe digit was a member of the memory set (‘‘target’’) or not (‘‘foil’’). They were instructed to respond as quickly and accurately as possible and were given a bonus of 5 cents for each correct response. This bonus was provided after completion of the scan. Within each block, half of the items were targets and the other half were foils. For each participant, target- and foil-button responses were randomly assigned to the right or left thumbs. The stimuli were projected onto a screen positioned on the head coil. Each of 3 runs contained 2 blocks of each of the 3 load conditions, presented in pseudorandom order, with the blocks of each load condition alternating with fixation (baseline) resting periods. Each run lasted 6 min.

The stimuli and responses were presented and collected using E-prime software (EPrime v1.1, Psychology Software Tools, Inc., Pittsburg, PA). Four participants (three patients and one control) were excluded from further analysis, because they completed a block with less than a 75% accuracy rate and/or with more

than 6 probes not answered within a block. Please see [3] for more information and a schematic depiction of the SIRP protocol.

SI 1.3 Image acquisition and processing

The T1-weighted structural brain scans at each of the four sites were acquired with a coronal gradient echo sequence: TR=2530 ms for 3T, TR=12ms for 1.5T; TE=3.79 for 3T, TE=4.76ms for 1.5T; TI=1100 for 3T; Bandwidth=181 for 3T, Bandwidth=110 for 1.5T; 0.625×0.625 voxel size; slice thickness 1.5 mm; FOV, 256×256×128 cm matrix; FOV=16 cm; NEX=1 for the 3T, NEX=3 for the 1.5T.

For all sites, functional images were acquired by using single-shot echo-planar imaging with identical parameters [orientation: AC–PC line; number of slices = 27; slice thickness=4 mm, 1-mm gap; TR=2,000 ms; TE=30ms (3T) or 40ms (1.5T), FOV=22cm; matrix 64×64; flip angle=90°; voxel dimen sions = 3.44×3.44×4 mm]. Cross-site calibration and reliability of these acquisition sequences for each scanner and the experimental set up for functional imaging as well as potential site and scanner differences were investigated prior to the study [4,5]. Additionally, test-retest reliability of functional and structural imaging data from ten MCIC subjects, who were all scanned at all four sites, was analyzed. Results showed that, even with different scanner manufacturers and field strengths, activation variability due to site differences is small compared to variability due to subject differences [4–6].

Structural data were analyzed using FreeSurfer. Within the validated and complex surface reconstruction algorithm for structural brain scans, white matter segmentations were produced, topological defects in the surface were automatically corrected and the white and gray matter boundary was tessellated. After intensity normalization, the gray/white matter and the gray/cerebrospinal fluid borders were detected at the location where the greatest shift in intensity defines the transition to the other tissue class [7,8]. Final surfaces were used to calculate cortical thickness at each vertex on the tessellated surface as the closest distance from the gray/white boundary to the gray/cerebrospinal fluid boundary [9]. Segmentation and surface reconstruction quality were assured by manual inspection of all raw MRI volumes, segmented volumes in three planes and pial as well as inflated volumes.

Functional images were registered to a high-resolution T1 image of the same subject (using a new algorithm called Boundary-Based Registration [10]) and to the standard space defined by the MNI-152 atlas. We did this by first registering the T1 images to the standard brain using FLIRT [11,12] and then composing the functional-

to-T1 and T1-to-standard registrations. We then fit a general linear model to the fMRI time course at each voxel in a whole brain model to estimate the average activation during the three loads of the probe condition in all trials. The magnitude of each Contrast Of Parameter Estimate (COPE), along with an estimate of its variability derived from model residuals, was passed to a second-level fixed effects analysis to combine COPE's from separate runs, yielding a composite T-statistic map for each contrast of interest for each subject.

Quality assurance steps for functional images included checks for whole-brain coverage of brain masks, motion and global mean intensity outlier timepoints, alignment of structural and functional scans, and registration problems (Epi to T1 and T1 to template). Outlier time frames in each fMRI data time series (detected using the artefact detection tools (ART) [13]) were defined by: *(i)* Global mean image intensity that differed by more than 3 standard deviations from the mean of the entire series of time frames in a scan, *(ii)* Displacement due to motion by more than 1 mm in the x, y or z direction relative to the previous time frame or *(iii)* Rotation due to motion by more than 0.1 rad around any of the three axes relative to the previous time frame. We removed the outlier time frames through the use of nuisance regressors in the linear model. In the case of runs where more than 15% of the time frames were flagged as outliers, the entire run was dropped from the analysis or the subject had to be excluded.

SI 1.4 Quality control measure for rs12807809 and rs12541

	**Genomic location**	**Genotyping rate (%)**	**GeneCall score**	**HWE (p-value)**			**LD (r^2)**
				overall	in patients	in controls	
rs12807809	chr11:124111495	100	0.84	0.37	0.34	0.79	0.05
rs12541	chr11:124128702	100	0.78	0.07	0.5	0.03	

SI Table S1: Quality control measure for rs12807809 and rs12541. Genomic location and quality control measure for rs12807809 and rs12541.

SI 1.5 Allele frequencies across populations

	**Hapmap sample**			**MCIC sample**		
	african ancestry	non-african ancestry	p-value	african ancestry	non-african ancestry	p-value
rs12807809 (C)	0.28	0.20	0.248	0.33	0.21	0.102
rs12541 (C)	0.75	0.23	<0.001[a]	0.61	0.21	<0.001[a]

SI Table S2: Allele frequencies across populations. Minor allele frequencies of rs12807809 and rs12541 in people with African and non-African ancestry were compared using a chi-square test. Population differences were observed for rs12541, but not for rs12807809. Hapmap results were confirmed in the MCIC sample. [a] significant based on a chi-square test.

SI 1.6 Monte-Carlo simulation

The Monte-Carlo simulation included the following steps: (1) An initial vertex-wise threshold (VWT) was set to p=0.05 to form spatially contiguous areas of association (referred to as a cluster). (2) The likelihood that a finding (cluster) of this size and magnitude (difference in thickness as specified by the VWT) would appear by chance, i.e. when using repeated random sampling, was tested using Monte-Carlo simulation with 10,000 repeats. This resulted in cluster-wise probabilities (CWP), which are reported using p-values throughout the results section.

SI 1.7 Estimates of cortical thickness and activation indices

We estimated the actual cortical thickness in millimeter (mm) in the identified cluster by labeling the cluster region as a region-of-interest (ROI), mapping it back on to each individual subject's unfolded surface by applying the same algorithm that morphed each subject's unfolded surface to the average spherical surface representation and extracting the average thickness for each individual.

Dorsolateral prefrontal cortex (DLPFC) ROI was derived from FreeSurfer cortical parcellations as described previously [14]. We obtained indices of activation for the DLPFC using the Contrast Of Parameter Estimates (COPEs) obtained from the second-level fixed-effects analysis for each participant. We applied an additional functional mask, based on the COPE of all loads (load 1, load 3 and load 5) versus fixation exceeding a threshold of Z = 2.3 and extracted the mean percent signal change (mean %Δ), defined as the mean COPE of all working memory loads.

SI 1.8 Power Calculations

SI Figure S1 displays the effect size-by-power relationship for the functional analyses. Rs12541 accounted for 8.9% of total variance of mean cluster activation (R^2 change after accounting for the effect of diagnosis and tesla field strength), corresponding to f^2=0.09 and a power of 99%.

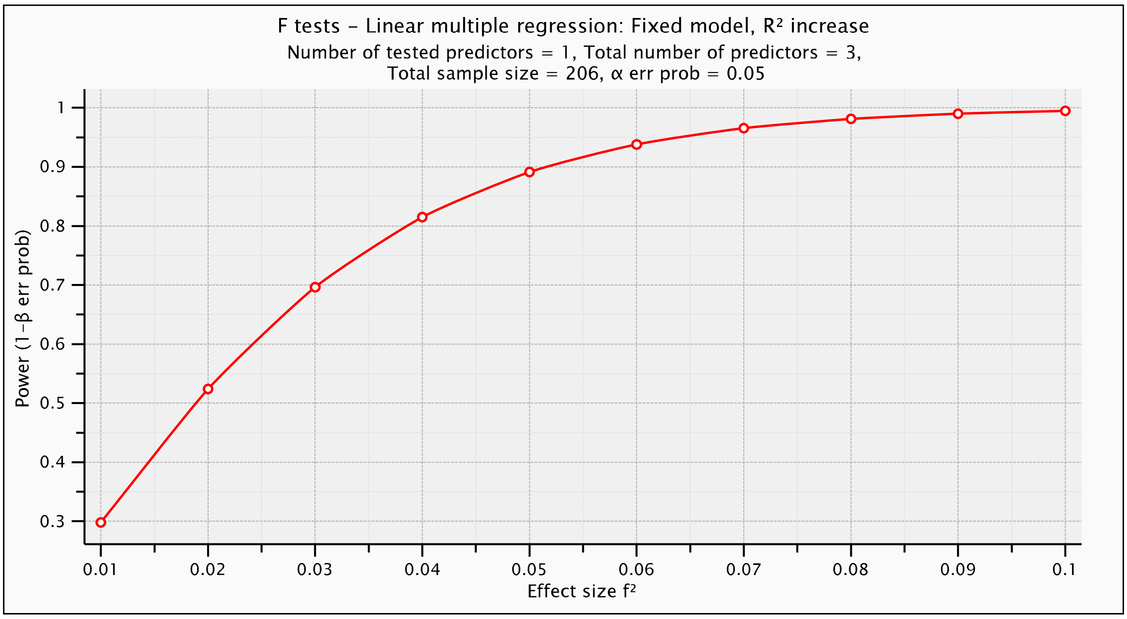

SI Fig S1: Power analysis of fMRI models.

Rs12807809 accounted for 3.1% of total variance of mean cluster thickness (R^2 change after accounting for the effect of diagnosis, tesla field strength, age and gender), corresponding to f^2=0.039 and a power of 85%.

SI 2.1 Statistical Assessment of Neuroimaging Findings

**Cluster Index**	**Z/F**	**x**	**y**	**z**	**Cluster-wide p-value**	**Cluster size (in voxels)**	**Area**
**whole-brain fMRI: rs12541**							
whole sample							
1	3.91	-26	38	34	0.0021	821	L DLPFC
2	3.96	-32	-26	14	0.00088	928	L insula
participants of non-African ancestry only							
1	3.67	-32	4	46	0.000603	973	L DLPFC
2	4.08	4	14	28	0.00386	748	R cingulate gyrus
**whole-brain cortical thickness: rs12807809**							
whole sample							
1	-3.095	14.4	-65.8	14.3	0.0227	1261	R pericalcarine gyrus

SI Table S3: Results of functional and structural imaging models for rs12541 and rs12807809 respectively. Abbreviations: Z, maximum z-statistic within the cluster; x/y/z, standard space coordinates of local maxima in mm.

SI 2.2 Additional fMRI Model

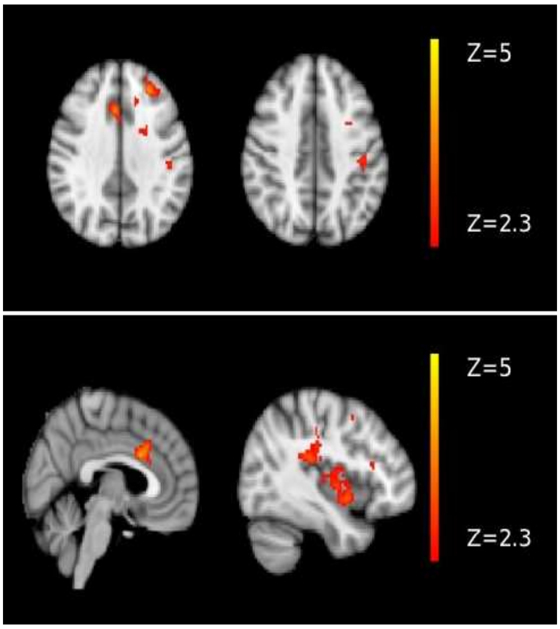

SI Fig. S2: Additional Model. Increased activity for rs12541 T/T homozygotes compared to C carriers could be replicated in a subsample controlled for population admixture by excluding participants of African ancestry. Results were cluster-corrected and controlled for diagnosis and scanner field strength. The z-values are represented according to the color code.

SI 2.3. Haplotype Analyses

**rs12541-rs12807809 haplotype**	**beta**	**t-statistic**	**p-value**
fMRI ROI			
omnibus test	-	15.6	0.00138
CC	-0.0746	4.86	0.0287
CT	-0.0924	11.4	0.000863
TT	0.0474	5.89	0.0161
cortical thickness ROI			
omnibus test	-	8.26	0.041
TC	-0.0669	6.07	0.0144

SI Table S4: rs12541-rs12807809 haplotype analysis results.

References

1. Andreasen N (1987) The diagnosis of schizophrenia. Schizophr Bull 13: 9–22.

2. Woods SW (2003) Chlorpromazine equivalent doses for the newer atypical antipsychotics. J Clin Psychiatry 64: 663–667.

3. Roffman JL, Gollub RL, Calhoun VD, Wassink TH, Weiss AP, et al. (2008) MTHFR 677C --> T genotype disrupts prefrontal function in schizophrenia through an interaction with COMT 158Val --> Met. Proc Natl Acad Sci USA 105: 17573–17578. doi:10.1073/pnas.0803727105.

4. Jovicich J, Czanner S, Greve D, Haley E, van der Kouwe A, et al. (2006) Reliability in multi-site structural MRI studies: effects of gradient non-linearity correction on phantom and human data. Neuroimage 30: 436–443. doi:10.1016/j.neuroimage.2005.09.046.

5. Jovicich J, Czanner S, Han X, Salat D, van der Kouwe A, et al. (2009) MRI-derived measurements of human subcortical, ventricular and intracranial brain volumes: Reliability effects of scan sessions, acquisition sequences, data analyses, scanner upgrade, scanner vendors and field strengths. Neuroimage 46: 177–192. doi:10.1016/j.neuroimage.2009.02.010.

6. Yendiki A, Greve DN, Wallace S, Vangel M, Bockholt J, et al. (2010) Multi-site characterization of an fMRI working memory paradigm: reliability of activation indices. Neuroimage 53: 119–131. doi:10.1016/j.neuroimage.2010.02.084.

7. Dale AM, Fischl B, Sereno MI (1999) Cortical Surface-Based Analysis: I. Segmentation and Surface Reconstruction. NeuroImage 9: 179–194. doi:10.1006/nimg.1998.0395.

8. Fischl B, Sereno MI, Dale AM (1999) Cortical Surface-Based Analysis: II: Inflation, Flattening, and a Surface-Based Coordinate System. NeuroImage 9: 195–207. doi:10.1006/nimg.1998.0396.

9. Fischl B, Dale AM (2000) Measuring the thickness of the human cerebral cortex from magnetic resonance images. PNAS 97: 11050–11055. doi:10.1073/pnas.200033797.

10. Greve DN, Fischl B (2009) Accurate and Robust Brain Image Alignment using Boundary-based Registration. Neuroimage 48: 63–72. doi:10.1016/j.neuroimage.2009.06.060.

11. Jenkinson M, Smith S (2001) A global optimisation method for robust affine registration of brain images. Med Image Anal 5: 143–156.

12. Jenkinson M, Bannister P, Brady M, Smith S (2002) Improved optimization for the robust and accurate linear registration and motion correction of brain images. Neuroimage 17: 825–841.

13. Whitfield-Gabrieli S, Thermenos HW, Milanovic S, Tsuang MT, Faraone SV, et al. (2009) Hyperactivity and hyperconnectivity of the default network in schizophrenia and in first-degree relatives of persons with schizophrenia. Proc Natl Acad Sci USA 106: 1279–1284. doi:10.1073/pnas.0809141106.

14. Ehrlich S, Morrow EM, Roffman JL, Wallace SR, Naylor M, et al. (2010) The COMT Val108/158Met polymorphism and medial temporal lobe volumetry in patients with schizophrenia and healthy adults. Neuroimage 53: 992–1000. doi:10.1016/j.neuroimage.2009.12.046.
